# Longevity leap: mind the healthspan gap

**DOI:** 10.1038/s41536-021-00169-5

**Published:** 2021-09-23

**Authors:** Armin Garmany, Satsuki Yamada, Andre Terzic

**Affiliations:** 1 Center for Regenerative Medicine, Marriott Family Comprehensive Cardiac Regenerative Medicine, Marriott Heart Disease Research Program, Van Cleve Cardiac Regenerative Medicine Program, Mayo Clinic, Rochester, MN USA; 2 Department of Cardiovascular Medicine, Mayo Clinic, Rochester, MN USA; 3 Mayo Clinic Alix School of Medicine, Regenerative Sciences Track, Mayo Clinic Graduate School of Biomedical Sciences, Mayo Clinic, Rochester, MN USA; 4 Division of Geriatric Medicine and Gerontology, Department of Medicine, Mayo Clinic, Rochester, MN USA; 5 Department of Molecular Pharmacology and Experimental Therapeutics, Mayo Clinic, Rochester, MN USA; 6 Department of Clinical Genomics, Mayo Clinic, Rochester, MN USA

**Keywords:** Geriatrics, Quality of life

## Abstract

Life expectancy has increased by three decades since the mid-twentieth century. Parallel healthspan expansion has however not followed, largely impeded by the pandemic of chronic diseases afflicting a growing older population. The lag in quality of life is a recognized challenge that calls for prioritization of disease-free longevity. Contemporary communal, clinical and research trends aspiring to extend the health horizon are here outlined in the context of an evolving epidemiology. A shared action integrating public and societal endeavors with emerging interventions that target age-related multimorbidity and frailty is needed. A multidimensional buildout of a curative perspective, boosted by modern anti-senescent and regenerative technology with augmented decision making, would require dedicated resources and cost-effective validation to responsibly bridge the healthspan-lifespan gap for a future of equitable global wellbeing.

## Healthspan-lifespan gap

**Fig. 1:**
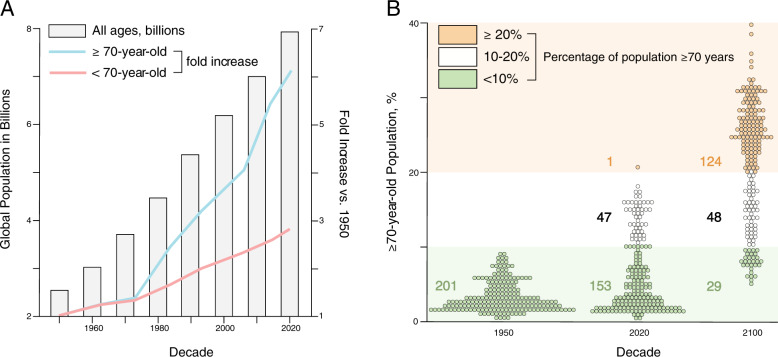
Global aging demographics. **A** The world population continues to grow and has reached nearly 8 billion people (bars). A preeminent increase has occurred in those older than 70 years of age (blue line), outpacing those younger than 70 years of age (pink line). **B** While in 1950 a ‘youthful’ age distribution (green) typified all geographies, by 2020 a fourth of the globe had transitioned to an ‘aging’ structure (white). Forecasts for 2100 imply that over 80% percent of all territories/areas will exhibit an ‘aging’ or ‘advanced aging’ composition (orange). Percent of population ≥70 years of age is stratified and color-coded in <10% (green), 10–20% (white), and ≥20% (orage) strata.

The world population has tripled^1^, from 2.9 billion in 1950 to 7.8 billion in 2020 (Fig. 1A). The average life expectancy—a benchmark of population health—has risen from 47 to 73 years of age in these seven decades, a 26-year expansion^1^. This remarkable trajectory in human longevity has generated a redistribution in demographic structure underpinned by a disproportionate surge in those over 70 years of age (Fig. 1A). Consequently, the number of countries with more than one-fifth of their population composed of those over 70 years of age continues to grow (Fig. 1B). This transition in aging demographics hinders global vitality^1^. Notably, the societal triumph of longevity is plagued with debilitating morbidity, accentuated towards the end of life.

**Fig. 2:**
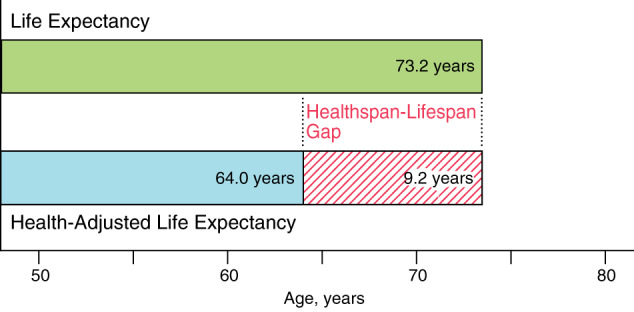
Healthspan-lifespan gap. Lifespan is the total number of years lived by an individual. Healthspan is the number of disease-free years lived. Life expectancy and health-adjusted life expectancy are population-level measures of lifespan and healthspan, respectively. A gap of 9-year is deduced from comparing 2020 data for median probabilistic projection of life expectancy and health-adjusted life expectancy.

Indeed, there is a recognized gap between lifespan, i.e., the total life lived, and healthspan, i.e., the period free from disease^2^. Using health-adjusted life expectancy, that considers life expectancy, years lived with disability, and premature death from disease^3^, the healthspan-lifespan gap is estimated at around 9 years (Fig. 2). This gap appears refractory to current practice paradigms. In fact, one-fifth of an individual’s life will be lived with morbidity^4^. Extending lifespan alone without delaying disease onset and/or reducing disease severity would actually aggravate the healthspan-lifespan gap. A guiding principle in addressing the healthspan-lifespan gap is in achieving health as “a state of complete physical, mental and social well-being and not merely the absence of disease or infirmity” per the World Health Organization (WHO). In this regard, integration of scientific breakthroughs with public and social programs is paramount towards success in extending a healthy lifespan. Learning from infectious disease control, remarkable success with river blindness required discovery of the anti-parasite ivermectin (awarded the 2015 Nobel Prize) and its broad dissemination accelerated by a drug donation program, achieving over 4 billion treatments and reaching 300 million people/year. Thus, to ‘compress morbidity’ and ensure the fundamental right to wellness, healthspan restoring strategies must evolve in unison of scientific, medical and social innovation.

## Disease and frailty challenge

Lifelong (also referred as “chronic” or “non-communicable”) diseases are the leading cause of mortality and disability worldwide^5,6^. Collectively, chronic diseases are responsible for 40 million or 71% out of 56 million annual deaths globally, and 79% of all years lived with disability^7,8^. Four common conditions, namely cardiovascular diseases, cancer, diabetes, and chronic respiratory diseases, account for 80% of chronic disease related deaths^9^. The imposed socioeconomic burden is estimated to represent a $47 trillion loss over the last two decades^10^. Fifty-eight percent of chronic disease-related mortality occurs in persons over 70 years of age. This growing age segment thus warrants special attention.

Age-associated outcomes are profoundly aggravated by frailty, a multisystem decline characterized by increased vulnerability. Frailty and associated geriatric syndromes are under-recognized despite engendering poor quality of life, disability, falls, hospitalization, long-term care, and mortality^11,12^. Assessment instruments use features of the “fraility phenotype” (weakness, slow gait speed, low physical activity, exhaustion, and unintentional weight loss) and “fraility index” (accumulative deficit) to identify and quantify frailty^12,13^. Present in around 25% of those older than 80 years of age and increasing in prevalence amongst younger age segments, frailty is accentuated by poor lifestyle choices and disproportionately affects those of lower-socioeconomic status and women, impeding equitable healthcare^14–16^. Responsible action plans should thus help re-design life in aging, aspiring to achieve quality with quantity.

## A global response

**Fig. 3:**
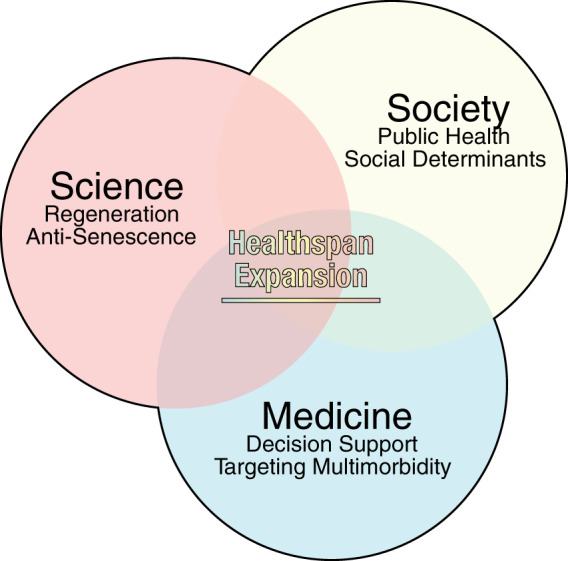
Healthspan extending toolkit. Healthspan extending strategies are comprehensive, relying on the unison of social, clinical and scientific programs. Societal initiatives include public health promotion and targeting of social determinants. Augmented decision making, harnessing multimodal datasets, has enhanced clinical management in the elderly. Breakthroughs in the anti-senescence and regenerative arsenal aim at curative solutions.

A series of programs, catalyzed by the United Nations (UN) General Assembly resolution 265, have been launched to lessen the escalating burden of non-communicable diseases. In concert with the UN Sustainable Development Goals^17^, WHO outlined 2025 targets for mortality reduction in 30–70-year-old individuals^18,19^. In parallel, professional healthcare organizations have stressed the relevance of disease-free life, exemplified by the American Heart Association 2030 Impact Goal set to lengthen by three years health-adjusted life expectancy^20^. Moreover, WHO has proclaimed 2021–2030, a decade of healthy aging^21^. Healthspan-centered actions will require an increasingly concerted, multidimensional effort that utilizes public health initiatives, acts on social determinants of health, and capitalizes on emerging technologies to equitably add value to senior life. Highlighting a multidimensional strategy for measurable goals, osteoporosis management combines diet and lifestyle interventions^22^, evidence-based screening^23^ and cost-effective therapy^24^. Accordingly, progress towards healthy longevity is further outlined below leveraging a communal, clinical, and research intersection (Fig. 3).

## The communal dimension

### Public health initiatives

Public initiatives, incorporating nutrition, water access, hygiene, vaccination, and antibiotics, have advanced primal disease prevention and reduced mortality. Furthermore, addressing modifiable risk factors, namely excess weight, physical inactivity, smoking, and poor diet, would prevent 80% of deaths from non-communicable diseases, corresponding to 57% of all deaths^25^. For conditions associated with non-modifiable risk factors (e.g., gender, genetic make-up), management goals include delay of disease onset and/or mitigation of disease severity. Enduring public initiatives are a recognized prerequisite for realizing healthy aging (Fig. 3). Large scale approaches rely on measurable multidimensional goals to modify the social and physical environment through banning public smoking, enforcing nutrition labeling, and regulating advertising and industry^26,27^. Tobacco use is a distinctive, avoidable risk underlying 8 million deaths per year, and is a declared ‘global health emergency’^28^. Five billion people worldwide are now covered by WHO-led MPOWER control measures encompassing use, prevention policies, protection from tobacco smoke, cessation programs, danger warning, bans on advertising, and raising taxes. Thus, emphasis on early prevention and optimization of public architecture that influence health decisions is essential^29^.

### Social disease determinants

In concert with influencing health behaviors, addressing the social basis of disease^30^ is vital. Childhood adversity, social alienation, maladaptive socioeconomic status, and compromised healthcare access are all associated with health inequality and reduced lifespan^31,32^. The level of attained education, work environment, prevailing wages, labor schedule, and reliability of work have serious bearing on lifestyle choices imposing disease risk^33,34^. Thus, reconciling and ameliorating structural underpinnings are critical to promote equitable health in the era of lifelong multimorbidity and population aging (Fig. 3). Correcting for social causes, in accord with public health initiatives, complements care strategies for the elderly, all essential in reducing disease burden. Collectively, interventions aimed at public and social determinants mitigate disease risks and lead to disease avoidance, namely primary prevention. Limiting poor outcomes of existing health issues, i.e., secondary, and tertiary prevention, demands additional clinical and research efforts.

## The clinical dimension

### Targeting multimorbidity

Addressing degenerative diseases in the elderly is a recognized priority of healthcare systems (Fig. 3), as over half of individuals over the age of 70 present with chronic multimorbidity. Deciphering disease pathobiology has led to new therapeutic avenues. Case in point, cancer therapies have been upgraded using biomarkers that inform personalized management^35–38^. Identification of tyrosine kinase inhibitor-responsive epidermal growth factor receptor variants in a subset of non-small cell lung cancer, and over-expression of human epidermal growth factor receptor 2 in breast cancer, offer guidance for molecular trait-refined individualized treatments^39,40^. In the elderly, precision biologics as adjuvant therapy are improving cancer care protocols, particularly for those susceptible to chemo-toxicity^41^. Disease substrates have been unmasked using newer molecular systems analytics (e.g., transcriptome, epigenome, proteome, metabolome, lipidome, microbiome)^42^. In cardiology, suggested benefit of hyperlipidemic control has been tested in a contemporary cohort study demonstrating prevention of myocardial infarction and atherosclerotic disease even in centenarians treated with statins^43^. Senior individuals are however commonly excluded from clinical trials, necessitating careful assessment of safety and efficacy in real world practice^44^.

The Hippocratic tenet “first do no harm” embodies the enduring principle of medicine, underscored in the National Commission for the Protection of Human Subjects of Biomedical and Behavioral Research axiom ‘maximize possible benefits and minimize possible harms’. In the elderly, a point of vulnerability is the risk of unplanned hospitalizations provoked by serious adverse drug reactions^45^. Commitment to non-maleficence is highlighted in evidence-based guidelines, including the American Geriatric Society’s Beers Criteria which outline drugs likely to produce unwanted actions in the elderly^46^. Effective pharmacotherapy for geriatric patients requires careful consideration of over- and under-prescription^47^. Optimal therapy relies on vigorous pharmacovigilance and shared decision making^48^. Decoding of the human genome has promoted the development of pharmacogenomics, providing evidence for inherent variation in pharmacodynamics and pharmacokinetics. Best studied, carriers of certain cytochrome P450 2D6 variants exhibit altered metabolism of drugs for pain management, cancer, and depression, medications broadly prescribed in the elderly^49^. Moderating dosage or giving alternatives, guided by recipient genotype, can maximize therapeutic outcome. Recently, pilot studies have begun to determine the utility of implementing pre-emptive pharmacogenomics in the clinical setting with focus on patient benefit. Rollout of pharmacogenomics in practice can be done at point-of-care, or proactively to augment future therapy decision making, and would require decisive return on investment and effective integration into a clinically actionable care plan. Powered by expanding translational experience, science-driven advances in individualized care are positioned to improve disease management.

### Augmented decision support

The digital health revolution has led to the acquisition of massive clinical data in diverse populations. Evidence-based care guidance is improved through build-out of robust electronic health record systems^50^. Contemporary, high-capacity, real-time data processing for personalized decision making is further augmented by machine learning modalities^51^. Artificial intelligence is deployed at the bedside to enhance human-guided analytics for diagnosis, prediction, and management for at-risk populations in the era of high-definition medicine (Fig. 3). Computational modeling helps to identify adaptive therapy regimens which outperform standard protocols, as exemplified in cancer care for multiple myeloma and breast cancer^52–54^. Broadly, geriatric oncology programs, prioritizing education, clinical practice, research, and strengthening collaborations and partnerships, are rapidly evolving to further the quality of care for older adults^55^. These prototypes are applicable in equivalent solution plans across the non-communicable disease spectrum^56,57^. Collectively, artificial intelligence-guided platforms, albeit nascent, hold promise for managing complex conditions in the aging population.

## The research dimension

### Targeting senescent cells

Cellular senescence and stem cell exhaustion, in conjunction with prime pathological states, such as genomic instability, telomere attrition, and mitochondrial dysfunciton, are hallmarks of aging^58^. Accordingly, addressing depletion or dysfunction in resident progenitor pools, concomitant accumulation of senescent cell load, and/or sterile inflammation are all considered in improving healthspan (Fig. 3)^59–61^. Removal of unhealthy cells that secrete pro-senescent paracrine factors has recently gained attention as an anti-aging strategy with assessment of senotherapeutics to selectively kill senescent cells or suppress associated pathophenotypes^62,63^. Pilot trials with first-generation senolytics report a decrease in senescent cell load, reduced inflammation, and frailty alleviation^64^, yet establishing definitive efficacy needs additional research and development^65^. In parallel, ambitious marketing creates a hype, adding to expectations of an otherwise vulnerable population. While conceptually attractive, senolytics must achieve increased specificity for senescent cells and restrict local tissue impact to minimize unwanted effects.

### A regenerative paradigm

Innate homeostatic and regenerative capacities decline with aging^66^. Elderly care demands special consideration in minimizing or delaying irreversible outcomes. Case in point, a third to a half of patients that survived a myocardial infarction will develop heart failure with a 50% mortality within 5 years^67^. The evolving knowledge in regenerative sciences is offering tools to halt or reverse refractory disease progression, transforming the goals of disease management ‘from care to cure’^68–70^ (Fig. 3). A dedicated framework of regulatory science, quality control, and bioethics has been deployed for responsible translation of a versatile regenerative medicine toolbox into validated patient delivery^71^.

The clinical readiness of regenerative therapies is maturing in age-related disease. In particular, regenerative immunotherapies have enhanced cancer management options. Chimeric antigen receptors, expressed in immune cells to combat antigen-expressing cancer cells, have been adapted for autologous delivery in hematological malignancies^72^. To contribute to physical and psychosocial quality of life in cancer survivors, tissue reconstruction after life-saving resection surgery has been tested. Examples include airway transplant enhanced by cryopreserved allogeneic aortic graft in patients with end-stage tracheobronchial disease^73^, and vascularized lymph node transfer for lymphedema after breast cancer surgery and/or radiation therapy^74^. In parallel with optimization of regenerative biotherapies, advancements in clinical grade manufacturing and delivery methods are ongoing, exemplified in the optimization of cardiovascular stem cell use for heart failure^75,76^. While regenerative approaches show safety and signs of efficacy for aging-associated diseases, regenerative therapies are yet to be fully tailored for aging populations to maximize benefit^77–79^. In this context, regenerative rehabilitation embodies a synergetic integration of biomaterial science, physical therapy, and regenerative medicine, offering a proven exemplar for the build-out of comprehensive models of care^80,81^. As a liberator from frailty and disease, the regenerative paradigm of normative restitution seeks to protect the person’s identity and help reclaim health^82^. Achieving a desired level of function and quality of life necessitates aligning regenerative feasibility with the values, goals, and outlooks of each individual. Broader adoption would require the roll-out of approved regenerative solutions that are standardized, scalable, and accessible/affordable.

## Economic consideration

Aging imposed cost burden on society has accelerated major investments into the high technology industry to harness anti-senescent and regenerative modalities. Fueled by realized and anticipated market returns^83,84^, the ongoing translation from bench to bedside necessitates careful consideration of the value proposition surrounding emerging therapies in the context of extended longevity. Notably, anti-aging science increasingly aims at developing prophylactic and curative interventions, promoting a transition from traditional symptom mitigation in advanced, disabling disease toward early, proactive health management^85^. Enabling long-term cost-saving and cost-effective benefit, radical curative solutions could pay-off the initial high costs that compromise utilization and hinder wider adoption^86^. Case in point, pilot real-world experience suggests reduction in cost of care following the introduction of regenerative immunotherapies in the treatment of blood cancers, such as lymphoma in Medicare patients^87–89^. Furthermore, health economic simulation based on initial clinical experience with cell therapy in Parkinson’s disease supports realizable cost-saving within a decade in the treatment of early onset degenerative conditions^90,91^. Similarly, stem cell-derived ß cell therapy for diabetes has been predicted to provide cost-benefit over life-long insulin therapy within an 8-year horizon^92^. Notwithstanding, health economic evaluation of advanced therapies remains limited and inconclusive^93–95^. Validated evidence of non-inferiority or superior efficacy from large treated populations, cost-effective mass production across the supply chain, and delivery within guidelines-recommended standard-of-care would advance cost-effectiveness analysis, necessary for fiscally sustainable decision-making^96,97^. Ultimately, society must reach a consensus on the threshold to pay for the value associated with the added benefit of validated emerging therapy for adoption in daily practice.

## An equitable future

**Fig. 4:**
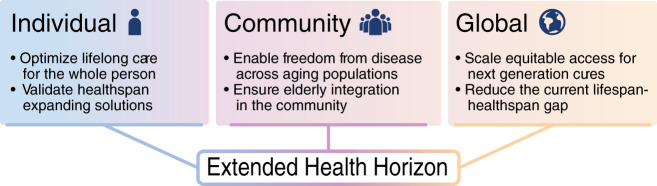
An equitable healthcare horizon. Healthy longevity mandates synchronized achievements at the individual, community, and global level. For each individual, holistic lifelong care must encompass validated healthspan expanding options. Freedom from disease and integration within the community must be ensured. Globally, in diverse populations, access to next-generation cures must be guaranteed to equitably reduce the healthspan-lifespan gap.

The steady rise in lifespan has been achieved but has not been met with a proportionate increase in healthspan. Paralleling the demographic transition is a multimorbidity and frailty burden, accentuated in the pandemic of chronic diseases. The compromised quality of life in the vulnerable elderly institutes a significant healthspan-lifespan gap, a formidable challenge confronting humanity. Contemporary hurdles across society, science, and medicine in healthspan extension include lack of transdisciplinary synergy, limited interdisciplinary resources dedicated to long-term common goals, and gaps in implementing high-definition science to medical practice in an equitable real world^98,99^. Accordingly, the deployment of integrated personal, communal, and global actions is a stated priority in order to deliver shared benefit across individuals and society-at-large (Fig. 4).

The SARS-CoV-2 viral outbreak and the ensuing COVID19 pandemic have tragically reminded us of the indispensable value of health, and unequaled impact amongst vulnerable populations^100^. The successful development of safe and effective vaccines, within a record timeframe, symbolizes the remarkable capabilities of modern science^101^. In contrast to acute harm, however, a gradually progressing danger tends to be under-recognized per analogy to the ‘boiling frog’ metaphor^102^. The insidious accumulation of chronic disease and frailty must engender disruptive innovation. Targeting the root cause at latent stages offers the prospect of implementing proactive, prophylactic actions. Beyond constraining disease symptomatology, disease-free outcome would require achieving enhanced intrinsic resilience against health-compromising stressors. Growing regenerative options offer opportunities to boost innate healing, and address aging-associated decline. Diverse aging populations are thus at the cusp of a promising horizon. Effective implementation of public health initiatives and amelioration of structural determinants will be accelerated by augmented decision-making and next-generation medical innovation driving new options. This outlook for extended well-being strives to achieve the ultimate goal that of health for all, to universally protect the longevity dividend.

## Data Availability

All datasests are from publically available data sources.
